# Facilitators and barriers to social distancing for young people during the COVID-19 pandemic

**DOI:** 10.1186/s12889-022-13325-3

**Published:** 2022-05-04

**Authors:** Emma Berry, Chris Jenkins, Sarah Allen

**Affiliations:** 1 School of Psychology, Queen’s University Belfast, Belfast, Northern Ireland; 2 Centre for Public Health, Queen’s University Belfast, Belfast, Northern Ireland; 3 Northern Ireland Statistics and Research Agency, Northern Ireland Civil Service, Belfast, Northern Ireland

**Keywords:** Health Behaviour, Physical Distancing, Young Adult, COVID-19

## Abstract

**Background:**

Social (or physical) distancing is an important transmission-prevention behaviour that has been endorsed to minimize COVID-19 transmission. This qualitative study explores the facilitators of and barriers to social distancing for young people during the COVID-19 pandemic, with recognition that young people represent a unique demographic group, with differing psychosocial needs and experiences to other age cohorts.

**Methods:**

Qualitative data was collected as part of a larger Qualtrics survey between July 28th 2020 and August 24th 2020. Eligible participants were young people living on the Island of Ireland, aged 16-25 years. The survey design was underpinned by the Capability, Opportunity, and Motivation model of behaviour change (COM-B). Semi-inductive thematic analysis was used to analyse comments collected via three free-text survey items. The COM-B model provided a thematic framework to organise subthemes extracted.

**Results:**

A total of *N* = 477 young people completed the survey, of which *N* = 347 provided comments for at least one of three free-text survey items. The majority of respondents lived in Northern Ireland (96%), the average age was 21 years, and most respondents were female (73%) and were students (81%). Key barriers identified included lack of environmental support for social distancing (lack of physical Opportunity to social distance), observing other people not social distancing (lack of social Opportunities supporting social distancing), and missing physical interaction from others (relating to the dissuading influence of automatic Motivational factors i.e. the influence of loneliness). Key facilitators included presence of clear and consistent environmental cues and availability of space to support social distancing (increasing physical Opportunity to social distance), increasing awareness and perceived consequences of risk of transmission (enhancing reflective Motivational factors i.e. perceived benefits (versus costs) of social distancing), and increasing opportunities to observe others’ adhering to guidelines (increasing social Opportunities supporting social distancing).

**Conclusions:**

These findings suggest that the actions and endorsement of peers and the physical design of environments have a key role in influencing social distancing behaviour among young people living in NI. The COM-B factors identified in this study can inform the development of tailored interventions using models such as the Behaviour Change Wheel. The findings of this study suggest that intervention functions based on peer modelling and physical environmental restructuring and enablement should be priortitised, however it is imperative that interventions are co-designed with young people to foster collaboration and empowerment.

**Supplementary Information:**

The online version contains supplementary material available at 10.1186/s12889-022-13325-3.

## Background

In response to the COVID-19 pandemic, social distancing guidelines were introduced in the United Kingdom (UK) and Ireland in March 2020 [1] and required fundamental changes in how people interact with each other. In the summer of 2020 (the time at which the present study was conducted), a number of non-pharmaceutical interventions (NPI) were in place in the UK and Ireland to mitigate the transmission of COVID-19. In certain contexts such as health and social care, hair and beauty, and hospitality settings, masks/face coverings were obligatory. However, during this time, for the majority of other public settings such as schools/universities, public transport, and shops, masks/face coverings were not yet enforced. Comprehensively required NPIs included maintaining hand and tissue hygiene (i.e. washing hands with soap for 20 seconds or more or using an antibacterial hand gel/spray, disposing of used tissues appropriately, and avoiding touching the eyes, nose, ears, and mouth) and social distancing behaviour. Social distancing (or physical distancing as it is more commonly referred to now) involves a wide range of strategies designed to minimise the spread of the virus by reducing contact between individuals, involving but not limited to, staying at home; reducing social contacts; and maintaining physical distance during interactions with other people [2, 3]. While guidance on whether individuals should remain one or two meters apart differs across countries, the key principle is that individuals limit their contacts and keep a physical distance between themselves and other people during any interaction.

Social distancing has been shown to be an important component of measures designed to reduce the spread and impact of COVID-19. In Northern Ireland (NI), early distancing measures were estimated to have reduced the R rate of transmissions from 2.8 to 0.8 [4]; a similar pattern seen in other parts of the United Kingdom (UK). A systematic review of early efforts to control the spread of COVID-19 additionally estimated that physical distancing of over one meter was important in reducing transmission (pooled adjusted OR 0.18, 95% CI 0·09 to 0·38) [5]. Furthermore, a review of studies on the effect of social distancing alone on H1N1 also found a median reduction rate of 23% of cases in the general population; combined with other measures this rose to a median reduction of 75% [6].

While young people (e.g. < 25) [7] who are deemed medically healthy may experience comparatively lower risk of serious effects of COVID-19, they play an important role in preventing its spread. Young people often have large social circles and are at a developmental stage in which social interactions and relationship building are considered important [7]. Young people may be more influenced by their peers, which may make adhering to social distancing regulations difficult should other members of their social groups not comply with guidelines [7]. In June 2020, in the UK and specifically NI, there were reports of large crowds of young people gathering on beaches and other public places raising concerns that young people were not adhering to social distancing guidelines, [8]. This raised concerns among public health bodies, prompting consideration of how best to support young people to engage in safer behaviour while socialising. Recent research has indicated specific challenges faced by young people during the pandemic related to isolation, loneliness and anxiety [7, 9], which may impact on behaviours related to social distancing.

Understanding the determinants of health behaviours is key in order to consider how to influence behaviour through targeted intervention approaches. Behavioural determinants refer to the psychological, social, environmental, and physical factors that collectively influence a particular behaviour or set of behaviours [10]. It has been proposed that behaviour change occurs when an individual has the physical and psychological Capability to make changes (this includes having the knowledge, skills, and physical ability to enact the behaviour), the Motivation to make changes (this includes evaluations about the behaviour (perceived benefits versus costs), intentions, and habits toward the behaviour, and the physical and social Opportunities to make changes (this includes being in contexts which cultivate the resources and social reinforcement required to enact the behaviour) (The COM-B model) [10]. Behaviour change occurs when Capability, Motivation, and Opportunity factors favour enactment of a new/alternative behaviour over other competing behaviours [10].. For example, an individual may be more likely to change their eating habits if they have adequate knowledge about nutrition and have the skills required to cook healthy recipes (Capability), if they perceive that eating healthier will improve their fitness and mental wellbeing (Motivation), if they can afford to purchase nutritional foods, and if family/friends support them with making the change (Opportunity).

The COM-B model thus provides scaffolding to explore how Capability, Motivational, and Opportunity factors collectively (and differentially) influence social distancing behaviour among young people. Identifying and understanding the interactions between these factors, and what influences them, is important to inform the design of interventions to support social distancing among young people; with the principle that particular behaviours are most likely to occur when Capability, Opportunity and Motivation are present.

Identifying the determinants of social distancing behaviour (relating to the COM factors) can inform the systematic selection of intervention functions and allied policy supports. The Behaviour Change Wheel (BCW) can be used as a guiding tool to support this [10, 11]. The COM-B is nested within the BCW. The BCW is an integrated model that provides broad guidance on 1. how to identify a target behaviour 2. how to identify the behavioural determinants (COM factors) 2. the selection of appropriate interventions to target the behavioural determinants 3. the selection of policy supports required to implement and sustain these interventions. In line with the BCW intervention development tool, a first step in understanding which interventions are appropriate to generating behaviour change (in this context we refer to the change in or maintenance of social distancing behaviour) is to identify the COM factors that the intervention needs to target [10, 11].

Given the retrospective and prospective importance of social distancing in reducing transmission of COVID-19, this paper uses the COM-B model as a theoretical lens to explore the psychological, social, and environmental factors which support (‘facilitators’) or thwart (‘barriers’) efforts to maintain a distance from others outside the household among young people living in NI or the RoI. The findings reported are based on a survey which was undertaken between July 28th 2020 and August 24th 2020 [12], as lockdown measures were being eased (this included the reopening of hospitality and leisure industries with physical distancing measures in place), and thus reflects on the societal and legislative context during this timeframe. As mentioned, during the point of data collection, masks/face coverings were not comprehensively required across all public settings, and social distancing was thus a key NPI in mitigating the transmission of COVID-19 during this period. This is important to bear in mind when considering the perceived importance of social distancing during this period and in the context of young people as they experienced greater opportunity to socialise in public and private settings, with the decrease in lockdown measures.

## Methods

### Design and setting

The cross-sectional survey collected anonymous quantitative data (via fixed response scales) and qualitative data (open text questions) remotely via Qualtrics software. The survey was open July 28th 2020 – August 24th 2020. The survey closed on August 24th as this marked the start of the process of returning to School/University/College which would introduce different contextual variance, thus impacting the interpretation of results. Ethics approval was obtained through Queen’s University Belfast on July 27th 2020 and the study is pre-registered on AsPredicted.org (reference: #45900). This study was also published as a preprint on Open Science Framework in June 2021: https://psyarxiv.com/yf6hk/

### Participants

Convenience sampling was used to recruit young people/adults aged 16-25 years living in NI/ROI on social media and via youth organisations, schools, and Higher Education institutions. Participants were given the opportunity to enter into a raffle to win a £100 Amazon voucher for completing the survey. As the primary analysis included frequency statistics (reported in full in Berry et al. [12]) and qualitative analytic methods, we aimed to recruit as many participants as possible to facilitate this analysis. Braun et al. [13] discuss an upper end of 100+ participants to support thematic analysis of online survey responses, and, while this is largely determined by the richness of the data and saturation, we aimed to recruit at least 100 participants. In summary, 96% of survey respondents were residents of Northern Ireland and 73% were female. The average age was 21 years with a standard deviation of 2.4 (range = 9). Most young people were living in their family home at the time of completing the survey and most respondents lived in households with 3-5 people (73%). One in 6 young people reported living with a chronic health condition, 9.6% reported having experienced symptoms consistent with COVID-19 (9.3% were unsure), and most young people reported that they were not shielding themselves or others in their household (95.9 and 81% respectively). See Table 1 for a breakdown of key demographics and health information of participants completing the survey. See Berry et al. [12] for full demographic information.

**Table 1:** Respondent demographic and health information

Demographic and medical information	Valid percentage of total sample (***N*** = 477)
**Country**	
Northern Ireland	96%
Republic of Ireland	4%
**Sex**	
Female	73%
Male	27%
**Age**	
16	3.7%
17	5.1%
18	8.5%
19	13.6%
20	12.2%
21	16.2%
22	14.2%
23	12.2%
24	5.8%
25	8.5%
**Living circumstances**	
Family Home	81%
Student accommodation	2%
Other rented accommodation	12%
Owned accommodation	2.5%
Other	2.5%
**Numbers per household/living accommodation**	
0	0.5%
1	1.1%
2	14.6%
3	21.2%
4	30.8%
5	20.6%
6	7.7%
7	2.7%
8	0.3%
9	0.3%
10	0.3%
**Do you live with a chronic health condition?**	
Yes	15.6%
No	84.4%
*Note. The majority of respondents reported living with Asthma or a mental health condition*	
**Have you ever been diagnosed with COVID-19?**	
Yes	1.1%
No	98.9%
**Have others in your household ever been diagnosed with COVID-19?**	
Yes	2.7%
No	97.3%
**Have any of your friends ever been diagnosed with COVID-19?**	
Yes	19.7%
No	80.3%
**Have you experienced COVID-19 symptoms?**	
Yes	9.6%
Unsure	9.3%
No	81.1%
**Are you shielding yourself?**	
Yes	4.1%
No	95.9%
Not applicable	–
**Are you shielding others in your household/living accommodation?**	
Yes	17.5%
No	81.1%
Not applicable	1.4%

### Materials and procedure

The online survey was developed in collaboration with members of the HSC Research and Development Division Behaviour Change Group [14], and was piloted by a representative group of young people to inform further adaptions to support relevance and understanding [15]. The survey items were guided by the COM-B model [10], to provide a theoretical framework a priori, and were deemed relevant given the applications of the model in relevant contexts [11].

The survey was divided into five sections: 1) COVID-19 knowledge and behaviour 2) social and environmental influences on social distancing 3) Attitudes and emotions about social distancing 4) Exploring views and experiences of social distancing 5) Demographics and COVID-19 exposure. Sections 1-3 and 5 were fixed response questions, which used yes/no or Likert scale answer formats (data reported in Berry et al. [12]). The three open text questions in section 4 included: Q1) ‘What things make it difficult to stick to the COVID-19 guidelines on social distancing?’ Q2) ‘What things help you to stick to the COVID-19 guidelines on social distancing?’ Q3) ‘What would make it easier to keep a distance when you see your friends or family outside your household?’

Potential participants accessed the survey via a link, which directed them to the information page and a series of consent statements. After providing consent participants were directed to the main survey and were provided with a debrief form upon completing the survey. Please note that sections 1-3 of the survey have been disseminated in a Public Health Agency report and therefore will not be reported again in this paper (see Berry et al. [12] for quantitative analysis of sections 1-3).

### Analysis

Descriptive statistics were used to capture the demographic composition of the sample, rates of COVID-19 related exposure, and to explore common barriers to social distancing through frequency statistics. A report has been produced for the Public Health Agency by members of the HSC R&D Behaviour Change Group, which presents the descriptive statistics and frequencies across responses in full and can be found online [12].

A combined approach to qualitative analysis was adopted to ensure that findings were methodologically sound, while also providing practically and clinically useful results. Reflexive thematic analysis [16, 17], framed by a subtle realist approach [18], was used to inductively code and develop subthemes. The qualitative analytic approach was informed by a review undertaken by McGowan and colleagues [19], which recommends that behaviour change theory should be applied flexibly in qualitative work, to ensure that codes and prospective themes are not limited by the framework applied. This enabled the primary qualitative analysts in the team (EB and SA) to inductively code, and develop subthemes liberally from the data. The analysis process followed the key steps outlined by Braun and Clarke [16] which includes transcription and familiarisation with the data, generation of initial codes, examining codes for themes, reviewing potential themes, defining themes, and formulating the report. The data was coded initially by SA and crosschecked by EB. Discussions took place to review codes, which led to the removal, addition, or amalgamation of codes. ‘Parent’ codes were reviewed and the units of meaning they represented helped to develop subthemes that remained bound to the source code but provided a higher degree of abstraction. This was an iterative process and a coding tree was used to ensure that the subthemes adequately captured the codes and were valid reflections of the data. Acknowledging the subjective nature of the analytic process, thoughts and observations were noted throughout the analysis and perspectives and interpretations were discussed during team meetings to support a comprehensive reflexive process [20].

The next phase of analysis followed a deductive thematic content analysis approach [21]. This consisted of a deductive mapping process in which the COM-B was used as a thematic framework to organise subthemes extracted. Subthemes extracted were categorised under the COM components of the COM-B model, where they were deemed to conceptually fit [19]. Data analysts SA and EB remained observant of subthemes that deviated from the COM-B framework. The COM-B thus provided a framework for overarching themes i.e. following the initial inductive coding and subtheme construction themes were assigned deductively. Both data coders are trained qualitative researchers and have a sound understanding of the COM-B model and its applications. EB, SA, and CJ contributed to the interpretation of themes and production of this report. Findings are reported in line with Consolidated criteria for reporting qualitative research guidelines in keeping with best practice (Tong, Sainsbury, & Craig, 2007) [22].

Deductive mapping of the more focused subthemes to COM components can facilitate public health professionals in the identification of intervention functions, policy categories, and behaviour change techniques according to the Behaviour Change Wheel [10]. While the purpose of this study was not to propose explicit intervention strategies, the mapped subthemes provide direction as to which intervention functions and policy supports can facilitate behaviour change in this context.

## Results

**Fig. 1:**
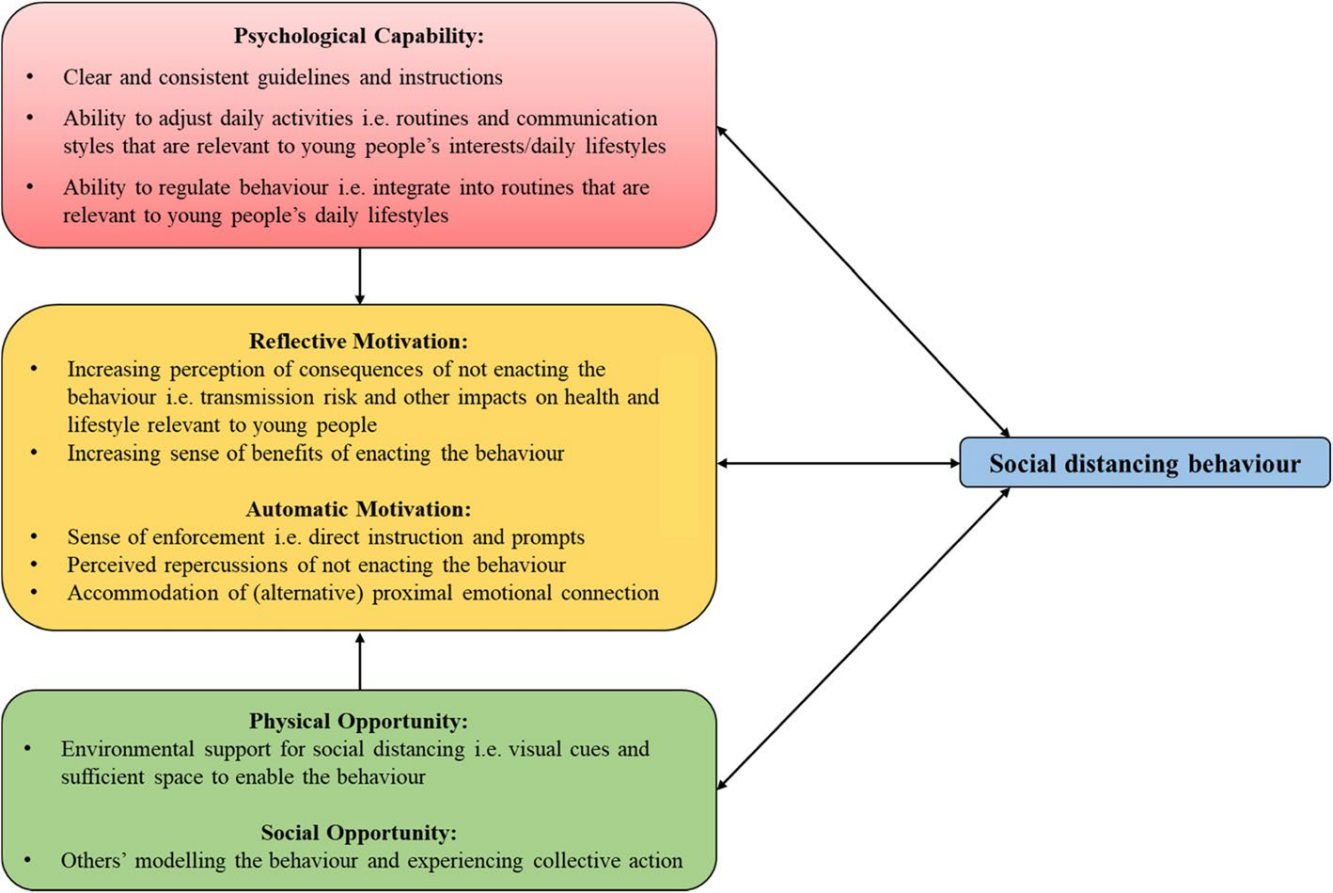
Determinants of social distancing behaviour in young people (adapted from COM-B conceptual model in Michie and colleagues [10])

A total of 477 young people completed the survey. Of the total *N* = 477 survey respondents, *N* = 347 respondents provided at least one free text response (this subsample did not differ significantly from the total sample across relevant demographic factors). In total *N* = 333/347 young people responded to Q1, *N* = 343/347 young people responded to Q2, and *N* = 335/347 young people responded to Q3. Thematic analysis of the each free text question led to the development of subthemes, which pertained to the perceived ‘barriers’ and perceived ‘facilitators’ of social distancing. Perceived facilitators was explored in the context of what is *currently* helpful to support social distancing *and* what would *further* help to support social distancing *more* (the latter is discussed in brief but full list of subthemes and supporting quotes can be found in Table 3). Subthemes extracted inductively are described under the Capability, Opportunity, and Motivation components, to which they have been conceptually mapped. Figure 1 provides a visual representation of how the subthemes extracted across each open-ended question fit within each COM construct. The figure, which is adapted from Michie and colleagues’ original COM-B conceptual diagram [2011], provides a summary of the key determinants of social distancing behaviour, collectively extracted through the analysis. The figure suggests that social distancing behaviour in young people is influenced by a specific set of factors, which fall within the Capability, Motivation, and Opportunity domains. Figure 1 further suggests that each of these factors are not mutually exclusive in the way that they influence behaviour, but rather their influence is interrelated and cumulative. For example, young people may be more inclined to social distance in various social settings, if there is available space to maintain a distance (physical Opportunity), if their peers are engaging in this behaviour (social Opportunity), if they perceive that there are consequences of not social distancing (Motivation), and furthermore if they have a sense of how to integrate social distancing in a typical social encounter i.e. behavioural regulation (psychological Capability).

### Barriers to social distancing (free-text question 1: perceived barriers)

Barrier-related subthemes mapped onto all three COM components, with the exception of physical Capability, which did not conceptually align with any of the subthemes generated (see Table 2).

**Table 2:** Barrier themes clustered under COM-B components and supporting quotes (Q1)

COM-B component	Subtheme	Sample quote
Psychological Capability	Lack of clear guidance about expected behaviour Challenge of regulating behaviour Effect of alcohol on ability to maintain behaviour	“Vague statements about distancing because people can interpret it in different ways” (participant 9) “It’s just culture that you hug people when you see them so sometimes you get caught up you forget.” (participant 199) “It’s difficult when others don’t do it or when any alcohol is consumed.” (participant 185)
Physical Capability	–	–
Social Opportunity	Actions of others as a disincentive	“People ignore the regulations and guidance. So if someone passes close to me then it defeats the purpose of me doing it if very few are watching their distance around everyone.” (participant 235)
Physical Opportunity	Lack of environmental support for social distancing	“Too many people in shops, too many people in general, not knowing what way to go in a shop” (participant 202)
Automatic Motivation	Absence of physical affection and contact	“Seeing your friends and family after so long and not being able to hug” (participant 26)
Reflective Motivation	Difficulty accepting the change in social behaviour’ Sense of low risk of transmission or contraction	“My family, friends, partner. Also, the fact i am sick of hearing the social distancing radio adverts constantly like propaganda. For me, the more i hear it, the less i listen.” (participant 307) “I want to be close to people I care about, to hug them, it’s hard if other people don’t as it feels pointless especially when transmission is very low” (participant 77)

Note. ‘-‘denotes absence of data to support the aligned COM component

#### Psychological Capability

Psychological Capability refers to an individual’s knowledge and skills to engage in the behaviour, and includes elements of self-regulation [10]. Three subthemes aligned with psychological Capability [42 total comments in total]: 1) ‘Lack of clear guidance about expected behaviour’, 2) ‘Challenge of regulating behaviour’, and 3) ‘Effect of alcohol on ability to maintain behaviour’. The first subtheme relates to the lack of confidence in and understanding of the formal guidelines around social distancing. There was uncertainty about what the instructions to social distance mean in reality, with messages described as “vague” (participant 9), and young people also felt like the messages were contradictory. Moreover, young people reported feeling confused because of the “Changing messages, scattered dissemination of information” (participant 271).

“Working as a key worker with friends, then meeting up with them outside work - it is nigh on impossible to socially distance in work and masks are only just coming in, why would we bother distancing if we go for food after work whenever we have just been closer than distancing in work?” (participant 144)

Regarding subtheme two, young people mentioned that it can be “easy to forget” to keep a distance from others while socialising because it’s not a habit (participant 25). This challenge of opposing entrenched social habits extends to the normative social behaviour people engage in while in the presence of others such as hugging others and shaking hands.

“Habits - i.e. being able to freely hug family members before, reaching for something in a shop where someone is also looking at the same item” (participant 352)

Subtheme three overlaps with subtheme two, capturing young people’s thoughts around alcohol consumption. Some young people felt that alcohol makes it more likely that people will revert to old habits and forget to distance.

“When people simply disregard the rules and get close to you. Alcohol also makes it difficult to stick to social distancing due to the loss of awareness.” (participant 198)

#### Social opportunity

Social Opportunity refers to the impact of an individual’s social and cultural environment on behaviour [10]. One subtheme extracted aligned with social Opportunity: ‘Actions of others’ as a disincentive’ [147 comments]. This was the most frequently reported barrier to social distancing and relates to observing other people not keeping their distance from others in public spaces. Being physically around other people who are not following the distancing rules makes it hard for young people to feel compelled to practice social distancing themselves. Seeing friends or other young people not keeping distance from others on social media is also discouraging as it implies that none of their peers are doing it.

“Pubs and restaurants not providing distancing at tables - have only met up with two friends since March, once for a walk and once to chat at a distance in a garden, and I left when the others went inside. Also, Instagram: everyone is posting pictures of themselves all over their friends at parties etc. and it’s so prevalent it sometimes makes me wonder if I’ve just imagined the whole pandemic? Why do I stick so strongly to the guidelines when nobody else I know seems to?” (participant 118)

#### Physical Opportunity

Physical Opportunity refers to an individual’s surroundings and physical resources that influence behaviour [10]. One subtheme extracted aligned with physical Opportunity [103 comments in total]: ‘Lack of environmental support for social distancing’. Many young people mentioned aspects of their physical environment that make it harder to distance. In particular, young people find it hard to keep a distance in small or busy spaces such as retail environments, friends’ homes, and workplaces.

“Small spaces in shops, corridors, work, friends houses etc... it isn’t always possible to effectively socially distance in certain places. Some people are also not very considerate of your own space” (participant 161)

Some young people also mentioned having to car share as an unavoidable barrier to keeping distance.

“Most of my friends don’t drive so they have to get into my car” (participant 83)

#### Automatic Motivation

Automatic Motivation refers to feelings, habits, and ‘innate dispositions’ that affect behaviour [10]. One subtheme extracted was categorized as automatic Motivation [71 comments in total]: ‘Absence of physical affection and contact’. The motivation to keep a distance from others was diminished due to young people missing physical affection and contact from friends and family. Young people missed being with friends/loved ones in-person and expressed feeling lonely because of the lack of in-person contact.

“I live in a rural area, and I was the loneliest I have ever been during lockdown. I was almost desperate to get back to Belfast to see my friends and boyfriend, who I hadnt seen in four months.” (participant 315)

#### Reflective Motivation

Reflective Motivation refers to the range of conscious evaluative or appraisal-based processes including perceptions toward the benefits (versus costs) of social distancing and perceived consequences of enacting (versus not enacting) this behaviour [10].. Two subthemes extracted aligned with reflective Motivation [21 total comments in total]: 1) ‘Difficulty accepting the change in social behaviour’ and 2) ‘Sense of low risk of transmission/contraction’. The first subtheme relates to young people’s evaluations of the change in social behaviour required to comply with transmission-prevention guidelines. This subtheme suggests that a number of young people found it difficult to integrate this new behaviour into their day-to-day life. For example, young people reported that keeping distance can feel unnatural and strange, which reduces their willingness and intentions to social distance. However, it should be noted that within this subtheme, there is also a sense of discomfort (of feelings) and difficulty regulating social distancing behaviour, which are experiences that conceptually align with the automatic Motivation and psychological Capability domains of the COM-B model.

“The pandemic being a new, unfamiliar situation in strange circumstances, causing it to feel unnatural to have to stay away from my friends etc., as I am used to closer contact with them - hugs, etc.” (participant 322)

Regarding the second subtheme, a smaller group of young people found it difficult to perceive the risk of not social distancing for reasons including disbelief of the infectiousness and seriousness of COVID-19 and feeling that measures taken such as forming a social “bubble of people to chat” meant they did not have to distance from friends (participant 344).

“The fact that none of my friends or I have ever caught it despite not social distancing. Can be hard to appreciate its importance” (participant 220)

### Facilitating social distancing (free-text question 2: perceived supports)

Subthemes related to facilitators to social distancing behaviour mapped onto all three COM components, with the exception of physical Capability, which did not conceptually align with any of the subthemes generated (see Table 3).

**Table 3:** Facilitator (existing supports) subthemes clustered under COM-B components and supporting quotes (Q2)

COM-B component	Subthemes	Sample quote
Psychological Capability	Clarity and consistency of guidelines and instructions Adapting (normal) lifestyle behaviour (include adapting usual forms of communication)	“Restrictions on everybody, clear and visible instructions” (participant 326) “Limiting how much time I actually spend with people outside my household. The more time you spend with people it’s easy to become used to it and forget about distancing” (participant 268)
Physical Capability	–	–
Social Opportunity	Actions of others as an incentive	“Having friends that are strict as well. If majority of people don’t, it makes you feel self conscious” (participant 15)
Physical Opportunity	Environmental support for social distancing: cues and space	“Rules in shops, tape on floors etc. that give me guides on where I should be standing.” (participant 159)
Automatic Motivation	Enforcement and perceived repercussions of not social distancing	“If it’s a legal requirement” (participant 1)
Reflective Motivation	Awareness of risk of transmission	“The knowledge that it is the right thing to do to keep people safe.” (participant 349)

Note. ‘-‘denotes absence of data to ort the aligned COM component

#### Psychological Capability

Two subthemes aligned with Psychological Capability [42 total comments in total]: 1) ‘Clear and consistent guidelines’ and 2) ‘Adapting (pre-pandemic) lifestyle behaviour’. Relating to the first subtheme, young people felt that keeping a distance from others in social settings is easier when public spaces like shops have clear instructions about how people are expected to behave and when there is “Consistent guidance from the government on the news and social media” (participant 52). Thus, clarity of instructions was important at a lower, rudimentary level e.g. shopping behaviour, however, there was also the sense that coherent messages from authorities was also important.

“Clearer views and instructions from the government as the people I know have varied knowledge of what’s the most up to date information.” (participant 284)

Some young people reported that adjusting their daily lifestyle by, for instance, “staying at home as much as possible” (participant 287) and only going out when necessary helps them to keep distance by avoiding situations where they need to ensure that they keep a distance from others. A small number of young people also mentioned that adjusting forms of communication with family and friends, for example talking to “friends through social media” and changing the setting of social meetings by “meeting up with friends in quiet parks” (participant 233) helps them to avoid close contact.

#### Social Opportunity

One subtheme extracted aligned with social Opportunity [55 comments in total]: ‘Actions of others as an incentive’. Many young people noted that it is easier to keep distance when others also keep distance, and it was felt that others supporting this behaviour by “acting as good role models” (participant 66) encouraged them to practice social distancing. The message underpinning this subtheme compliments the social opportunity barrier previously acknowledged.

“Seeing others distancing make me distance” (participant 124)

#### Physical Opportunity

One broad subtheme extracted aligned with physical Opportunity [146 comments in total]: ‘Environmental support for social distancing: cues and space’. This subtheme suggests that physical environment has a large impact on young people’s perceived ability to keep distance and their capacity to remember to keep distance. One element of this subtheme related to how young people felt that environmental cues and reminders and physical prompts facilitated their ability to keep a distance from others and reminded them to keep a distance from others.

“Stickers on the ground, spaced out tables when eating, reminders on social media/billboards etc” (participant 155)

Another aspect of this subtheme related to the need to have adequate space to practice social distancing, which suggested that young people felt that keeping a distance from others is easier when the physical environment aids this, for example by restructuring indoor spaces or meeting others in outdoor settings.

“Meeting outside, or in spaces/restaurants with good safety precautions and spacing” (participant 182)

#### Automatic Motivation

One subtheme extracted aligned with automatic Motivation [22 comments in total]: ‘Enforcement and perceived repercussions’. A number of young people also mentioned that they keep distance when they are made to. This primarily related to circumstances when (shop/other) staff enforce it or “implementing laws” (participant 168), in which case the risk of punishment is visible.

“when there are strict rules in shops/ businesses that easily instruct you what to do before entering. However I only actually do it if there is a member of staff at the door reminding everyone to use the soap dispenser.” (participant 321)

#### Reflective Motivation

One subtheme extracted aligned with reflective Motivation [95 comments in total]: ‘Awareness of risk of transmission’. Young people frequently mentioned an awareness of risk as a motivation to keep a distance from other in social situations. They reported feeling motivated by a drive to keep themselves or others safe, to avoid deaths and “helping stop the spread” (participant 92). Some said they kept distance because it was “the right thing to do from the news” (participant 228), which may relate to a number of factors including news reports of contagion and death rates, as well as news coverage on government guidelines around social distancing behaviour.

“Knowing I’m less likely to make myself/others sick” (participant 64)

A small group of young people also reported feeling more motivated to keep a distance from others in social situations after “Noticing that daily cases of covid are increasing” in their own “council area” (participant 251).

### Facilitating social distancing (free-text question 3: further supports needed)

The final question asked respondents what could be improved or put in place to further facilitate social distancing behaviour. Subthemes for this question were mapped onto all three COM components, with the exception of physical Capability, which did not conceptually align with any of the subthemes generated (see Table 4). Subthemes largely complimented the subthemes identified for current/existing facilitators, with regard to greater clarity of guidelines in different social circumstances, increased environmental supports, increased visibility of the supportive behaviour of others, the need for more enforcement of ‘rules’, and the importance of knowing the risks of contagion and contraction.

**Table 4:** Facilitator (*additional supports needed*) subthemes clustered under COM-B components and supporting quotes (Q3)

COM-B component	Subthemes	Sample quote
Psychological Capability	Clear and consistent guidelines Adjusting lifestyle and social activities	“Clearer guidelines. There are too many contradictions about!” (participant 177)
Physical Capability	Environmental supports to facilitate and encourage behaviour	“Perspex glass divisions in indoor spaces, areas marked with tape outside, maybe a technological reminder that youre standing too close to someone? Like an app” (participant 305)
Social Opportunity	Supportive beliefs and values of others	“If everyone had the same goal in mind. Lots of people don’t believe in the virus or in the prevention measures” (participant 130)
Physical Opportunity	–	–
Automatic Motivation	Enforcement of rules with repercussions	“More clear or strict guidelines, if it is necessary. If everyone is forced to do it, then it would be easier.” (participant 139)
Reflective Motivation	Increasing awareness of the benefits versus risks Sense that nothing (more) can help	“More public understanding of the severity of coronavirus. Most people are bored of it now and seem to act like it doesn’t exist anymore.” (participant 158) “Nothing it’s always going to be hard” (participant 68)

Note. ‘-‘denotes absence of data to support the aligned COM component

However, one subtheme, which aligned with reflective Motivation, conveyed a different perspective to any of the subthemes described previously [71 comments in total]: ‘Sense that nothing (more) can help’. The comments that led to the development of this subtheme tended to relate to the perspective that there is “nothing that can make it [social distancing] easier” and that distancing from others was “common sense” (participant 183), or indicated an uncertainty about what can further help or the sense that nothing can help.

“Nothing. To be honest I have no ideas on how it could be made easier.” (participant 317)

## Discussion

### Overview of determinants of social distancing in young people

This study explores the commonly experienced barriers to and facilitators of social distancing behaviour in young people predominantly from Northern Ireland, using the COM-B model to organise and make sense of the data. This study focuses on a sample of young people, primarily students, who recognise the supports needed to endorse social distancing behaviour, but who experience a range of factors that impede the extent to which they implement this behaviour in daily life. As reported in Berry et al. [12], while a large number of respondents reported that they social distance frequently, a substantial proportion reported that they do not. This is consistent with previous evidence indicating that adherence to social distancing guidelines is generally lower in younger populations [23, 24]. The frequency data reported in Berry et al. [12] suggests that actions of peers, forgetting to keep a distance from others, environmental constraints, and unhelpful emotions contribute to intentions to, or perceived ability to social distance (see Berry et al. [12]). Qualitative analysis of free-text survey responses provided the scope to further explore these observed frequencies.

A large number of responses in relation to the barriers and facilitators of social distancing revolved around physical Opportunity, and more specifically, the importance of context (e.g. place and occasion), visual cues (e.g. floor markings), and prompts (e.g. visual and verbal reminders) in helping young people to social distance. This is an important area to consider when designing interventions to prompt behaviour and can be particularly effective when combined with clear and simple guidance. As suggested by the current findings, social distancing behaviour among young people is influenced by the structure and organization of businesses. For example, pubs, restaurants, and shops not accommodating and/or not enforcing social distancing guidelines was a commonly reported factor among young people who took part in the study. Correspondingly, in the current study, visual cues and reminders, guidance from staff, and premises following guidelines were consistently highlighted as enablers for social distancing.

Observing the actions of peers or others’ not distancing was also a commonly raised challenge for young people who took part in the study. The powerful influence of social norms, and the innate desire to comply or confirm with the social distancing intentions and behaviour of peers, is also demonstrated by Martínez and colleagues [25]. Additionally, seeing friends/others posting photos on social media in which social distancing was absent was another important barrier to social distancing which specific messaging and social normative interventions could target. Moreover, the requirement to maintain physical distance from friends, partners, and family, and the emotional difficulties this caused, further contributed to the wider social challenges experienced by the young people who took part in the study. Certainly, evidence demonstrates the negative psychological impact of continued isolation for young people in particular since their life stage is more intensely shaped by their social environment (i.e. exposure to peers and learning environment) [26–28]. The reliance on distance learning, resulting in reduced social interaction and changes to the home environment, have contributed to the impact of quarantine measures in general on emotional, social, and behavioural outcomes in both children and young people [29, 30]. Longitudinal evidence demonstrates increased symptoms of depression and worsening quality of sleep among young people over the duration of the pandemic, which affirms the psychological impact of prolonged periods of quarantine in this demographic population [31]. Despite this, research suggests that young people express greater willingness and openness to discussing their emotional wellbeing now, than during pre-pandemic times – so there is an opportunity to engage with and empower young people to shape post-pandemic social systems and mental health support [32].

It is essential that future interventions and public health campaigns for young people appreciate young peoples’ experiences of lockdown and their current reality; utilising strategies to help young people navigate these challenging psychological and social dynamics. A number of young people who participated in the current study indicated that they found it difficult to accept the ‘reality’ of the pandemic, which, since the first lockdown ensued has resulted in drastic change to day-to-day life – some young people found it difficult to integrate the changes in social behaviour because it felt uncomfortable and unusual. It is notable that among the young people who took part in the study, there was an absence of reported feeling of invincibility to engage in life as they would have pre-COVID-19. With this in mind, it is understandable why young people in general find it more difficult to follow guidelines on social distancing if they are less able to accept the lifestyle changes and sacrifices advised.

For the most part, however, many respondents reported feeling worried about catching the virus or transmitting it to others. In fact, this increased awareness of risk was a commonly suggested enabling factor for social distancing in young people. Given the demographic information in the sample, the majority of young people lived in their family home, suggesting that they do not live alone and rather, are likely to live with parents/guardians. The demographic and health background of the sample suggests that young people (at the point data was collected) had little exposure to COVID-19 directly, however 1 in 5 young people reported that they were shielding a loved one and 1 in 5 also reported that their friends had shown symptoms of COVID-19. This suggests that the young people who participated in the study had concerns about contagion, particularly to close persons like family/people in their household, which corroborates the observation that risk awareness can enable social distancing.

Given the nature of the behaviour and the demographic population under study, it is perhaps not surprising that physical Capability was the only COM factor that did not conceptually align with any of the subthemes generated. Physical Capability can relate to abilities such strength, stamina, or physical skill/technique required to enact a behaviour. It could be argued that social distancing is not a particularly strength-, stamina-, or skill-demanding behaviour, and rather could be considered an inaction or a movement to avoid others. The findings suggest that, if young people have the space and environmental scaffolding to facilitate social distancing and are motivated to enact this behaviour; physical ability may be a less common barrier for this particular sociodemographic group. Although we cannot extrapolate this assumption to other demographic groups whose physical capabilities may have a bigger role in ability to distance from others.

#### Practical implications: considering intervention approaches

This study captures the experiences and perspectives of young people during an unforeseen and unfamiliar societal trauma. The survey aimed to capture the factors which thwart or support social distancing, however the content of themes highlight the wider psychosocial challenges experienced by this group, such as the absence of physical affection and feeling jaded by social structures. The results bare relevance for future challenges of maintaining physical distance, particularly as lockdown restrictions have eased and with consideration of future pandemic challenges. Using the Behaviour Change Wheel (BCW) as an extended theoretical framework [10], the barriers nested within each of the COM components can be targeted through a series of intervention functions and policy supports, which evidence suggests can support behaviour change. Figure 1 provides an overview of the key determinants of social distancing behaviour, which are categorised into the relevant Capability, Opportunity, and Motivation components. This figure can facilitate identification of potential intervention functions, which the BCW suggests, can target selected behavioural determinants [10].

The findings of this paper indicate that interventions intended to encourage social distancing must go beyond informing young people about the need to social distance (of which they demonstrate high levels of understanding already) but should focus on environmental and social enablers and barriers. The prevalence of responses focusing on physical and social Opportunity determinants of social distancing suggests that interventions targeting these factors should be prioritised. To address these determinants, based on the guidance of the BCW, intervention functions may include enablement and environmental restructuring (e.g. via tangible/visual cues in shops, facilitating space to distance, reminders to maintain a distance). Such intervention strategies can increase young people’s physical Opportunity to social distance, but may also facilitate behavioral regulation and habit formation (which, in turn, also target some of the psychological Capability and automatic Motivational factors summarized in Fig. 1). The physical design of our environment (i.e. the structure, layout, and aesthetics) influences how we interact with features and other people in that environment, which has been captured by studies demonstrating the effect of environmental restructuring on health and social behaviours in young people such as bullying [33] and alcohol intake [34]. Few studies have explored this in the context of social distancing. However Hagger and colleagues [35] suggest that the physical restructuring of spaces in a way that provides scaffolding on to how to enact social distancing, alongside consistent cues and reminders, can support social distancing behaviour and may eventually lead to habit-formation. Despite the speculated benefits of environmental restructuring, there is a dearth of evidence exploring this intervention function in the context of social distancing [35, 36] which raises an important gap in the pandemic behaviour change literature.

To address the social Opportunity determinants captured in Fig. 1, using the BCW as a guiding tool, we recommend peer modelling (i.e. peers demonstrating social distancing and leading by example) with embedded social incentives (i.e. encouragement from peer role models) to increase social oOpportunities that favour social distancing. Effective peer modelling of social distancing behaviour may consist of peer leaders in community settings or social media influencers demonstrating and promoting this behaviour, rather than politicising this behaviour through authoritarian messaging styles [37, 25]. Crucially, this intervention approach focuses on modelling of social distancing behaviour by individuals and communities who young people have respect for, trust, and who they can empathise with. This may include friends, club membership leaders (e.g. football coaches), and social media influencers [25, 37]. Peer modelling of social distancing behaviour can be demonstrated in-person or via social media live streams/reels/other visual posts. However, modelling of social distancing behaviour can also be demonstrated through other novel mediums such as health comics and animations. For example, evidence exploring the use of comics to promote of COVID-19 transmission-preventative behaviour suggests that comic art can be used to communicate health messages in a more accessible way, by creating fun, emotive, and memorable characters and narratives, which young people (along with other demographic groups) can identify and empathise with [38].

Beyond these recommendations, it is important to also note that changing behaviour is not just about ‘delivering the message’ to young people to try to convince them to do the right thing. Nor can we assume that young people are less invested or interested in public health advice. What is more important is that health communication is tailored in a way that resonates with and is relevant to young people. Public health practitioners will be more successful in influencing the actions of young people if they address the unique barriers identified for this important demographic group, adopting a holistic approach, which acknowledges the range of behavioural influences and considers the unique challenges that COVID-19 poses for young people. This requires having empathy for young people and ensuring that their needs and concerns are at the heart of the design of any intervention. Otherwise, engagement is likely to be low or short-lived. Local and international reports affirm the need to capture to voices of young people to help shape governmental decisions and policy to support engagement and also mitigate the long-term impact of COVID-19 for the younger generation, who are likely to be more harshly affected by the psychosocial and economic repercussions of prolonged periods of lockdown [9, 39]. Bonell and colleagues [37] highlight the importance of co-designing and piloting interventions targeting social distancing, which may entail hosting focus groups to inform the design of interventions and ethnographic work to explore how young people behave in day-to-day social encounters, which provide important insights relevant to the implementation of interventions in real contexts.

Additionally, it is important that the efforts made by young people are not undervalued and that incidents of young people not adhering to guidelines are not sensationalized by the media (e.g. scenes of young people gathering in public spaces, house parties), which, as conveyed by the social Opportunity subthemes, inadvertently reinforces the undesirable behaviour. In other words, the more that young people are provided with these *less helpful* role models (in contrast to the function of peer modelling intervention strategies discussed above), the *less* incentivized they will be to engage in social distancing [37, 40]. There is also a need to rebuild or maintain trust in civil society organisations, which may have decreased for young people (among other demographic groups) as result of unclear decisions, conflicting messages, and frustration [39]. This affirms the importance of tailoring content and delivery mediums of health communication to resonate with young people.

#### Limitations and future directions

This study was conducted during the graduated easing of the first lockdown during the summer of 2020 and thus is reflective of the thoughts, feelings, and behaviours, which occurred in this context. In particular, as mentioned earlier, at the time of data collection (Summer 2020), social distancing was a comprehensively advised NPI, while instruction to wear a face covering was enforced only in specific settings. Moreover, since the vaccine rollout commenced late 2020/early 2021, it is possible that perception of risk has decreased, thus influencing transmission-preventative behaviour generally [41]. While vaccine behaviour in itself is an important public health matter, we also need to consider how receipt of the vaccine has impacted willingness to maintain social distancing recommendations as the vaccine roll-out has progressed. In general, transmission-preventative and social behaviours have evolved since the time the study was conducted, therefore, the perspectives provided in this study are reflective of the context in which data was collected.

The findings from this study should therefore be seen as indicative of important trends, but further research to validate the findings is needed. In particular, through intervention-based research to evaluate the effectiveness of intervention functions such as environmental restructuring in the context of social distancing (perhaps through hypothetical experimental work initially and thereafter in contexts of coronavirus contagion where social distancing is advised/reintroduced). Further qualitative research would be additionally useful in deepening understanding of the themes presented within this study, since the computer-based survey format limited the extent to which responses could be expanded upon. This cross-sectional study also restricts the findings to a single snapshot of social distancing perceptions and behaviours at the time data was collected. Longitudinal work would provide the data to examine change in perceptions and behaviour overtime, which is valuable to measure the stability of these factors.

Moreover, respondents from the survey were almost exclusively from Northern Ireland. Even though many findings may be similar for young people in the Republic of Ireland, generalizing such findings should be treated with caution. Information on ethnicity was also not collected. Given the disproportionate impact of COVID-19 on BAME communities [42, 43], further research within cohorts of young people from minority ethnic groups is important. The average age of participants was 21 years old, so our study provides limited insight into the experiences of younger participants aged between 16 and 18 years old. Also, only 27% of respondents were male, again highlighting a group towards which future research may be targeted. Other research has indicated that social distancing adherence is lower in men [24], further indicating the importance of understanding responses and behaviours among younger men. Given the demographically and culturally homogenous sample, we cannot generalize the findings of this study to the wider population of young people. Therefore we recognise that there are limits to the extent to which we can advise on specific interventions targeting social distancing in different countries and sociocultural contexts.

## Conclusion

This study provides a novel glimpse of the determinants of social distancing among young people living in NI/ROI, which is visually captured in Fig. 1, providing information relevant for the design of targeted public health interventions using frameworks such as the Behaviour Change Wheel [10]. In particular, we discuss the importance of interventions which focus on harnessing the power of peer influencers and redesigning physical environments in ways that favour social distancing. Such approaches to social distancing messaging deviate from traditional authoritative health communication styles, and may be more effective at nudging or more explicitly encouraging social distancing behaviour in young populations. As lockdown measures have eased, it is important to understand current social distancing behaviours and intentions to maintain these where necessary in the longer-term, especially as the nature of the pandemic is inherently unpredictable and the emergence of new variants a potential risk. Given the pace of social change during the course of the pandemic, regular research should be conducted on how young people’s behaviours are changing in response to different social distancing requirements and legislative changes.

## Supplementary Information

**Additional file 1.**

## Data Availability

Data is available as a supplementary document.

## Abbreviations

UK: United Kingdom
NI: Northern Ireland
RoI: Republic of Ireland
COM-B: Capability, Opportunity, and Motivational model of Behaviour
BCW: Behaviour Change Wheel
COVID-19: SARS-CoV-2/Severe acute respiratory syndrome coronavirus 2
